# Using natural language processing to facilitate the harmonisation of mental health questionnaires: a validation study using real-world data

**DOI:** 10.1186/s12888-024-05954-2

**Published:** 2024-07-24

**Authors:** Eoin McElroy, Thomas Wood, Raymond Bond, Maurice Mulvenna, Mark Shevlin, George B. Ploubidis, Mauricio Scopel Hoffmann, Bettina Moltrecht

**Affiliations:** 1 School of Psychology, Ulster University, Coleraine, UK; 2 Fast Data Science, London, UK; 3 School of Computing, Ulster University, Belfast, UK; 4 Centre for Longitudinal Studies, University College London, London, UK; 5 Department of Neuropsychiatry, Universidade Federal de Santa Maria (UFSM), Avenida Roraima 1000, Building 26, office 1353, Santa Maria, 97105-900 Brazil; 6 Graduate Program in Psychiatry and Behavioral Sciences, Universidade Federal Do Rio Grande Do Sul, Rua RamiroBarcelos 2350, Porto Alegre, 90035-003 Brazil; 7 Mental Health Epidemiology Group (MHEG), UFSM, Santa Maria, RS Brazil; 8 Care Policy and Evaluation Centre, London School of Economics and Political Science, London, UK; 9 National Center for Innovation and Research in Mental Health, São Paulo, Brazil

**Keywords:** Retrospective data harmonisation, Harmonisation, Meta-analysis, Data pooling

## Abstract

**Background:**

Pooling data from different sources will advance mental health research by providing larger sample sizes and allowing cross-study comparisons; however, the heterogeneity in how variables are measured across studies poses a challenge to this process.

**Methods:**

This study explored the potential of using natural language processing (NLP) to harmonise different mental health questionnaires by matching individual questions based on their semantic content. Using the Sentence-BERT model, we calculated the semantic similarity (cosine index) between 741 pairs of questions from five questionnaires. Drawing on data from a representative UK sample of adults (*N* = 2,058), we calculated a Spearman rank correlation for each of the same pairs of items, and then estimated the correlation between the cosine values and Spearman coefficients. We also used network analysis to explore the model’s ability to uncover structures within the data and metadata.

**Results:**

We found a moderate overall correlation (*r* = .48, *p* < .001) between the two indices. In a holdout sample, the cosine scores predicted the real-world correlations with a small degree of error (MAE = 0.05, MedAE = 0.04, RMSE = 0.064) suggesting the utility of NLP in identifying similar items for cross-study data pooling. Our NLP model could detect more complex patterns in our data, however it required manual rules to decide which edges to include in the network.

**Conclusions:**

This research shows that it is possible to quantify the semantic similarity between pairs of questionnaire items from their meta-data, and these similarity indices correlate with how participants would answer the same two items. This highlights the potential of NLP to facilitate cross-study data pooling in mental health research. Nevertheless, researchers are cautioned to verify the psychometric equivalence of matched items.

**Supplementary Information:**

The online version contains supplementary material available at 10.1186/s12888-024-05954-2.

## Introduction

There is increased recognition that pooling data from different sources can help us to better understand and treat mental health problems [1]. Pooling data has statistical benefits (e.g. increased sample sizes), and it can also help uncover important contextual differences across cultures and time [2–4]. In the UK, initiatives such as the Catalogue of Mental Health Measures [5], CLOSER [6], Datamind [7], and the UK Longitudinal Linkage Collaboration (UK LLC) [8] have made it easier than ever for researchers to find and pool data from different sources. However, in practice most mental health research is conducted in measurement silos, and more often than not there are inconsistencies in how variables are measured across studies. Indeed, it has been estimated that close to 300 instruments have been developed to measure depression alone [9]. Self-report questionnaires, one of the most common approaches to measuring mental ill-health, can differ markedly on the types of symptoms they enquire about, even when supposedly measuring the same disorder [10]. Such heterogeneity in measurement can impede attempts to pool otherwise comparable datasets.

Retrospective harmonisation is an increasingly popular solution to this problem. This refers to the process by which data from different sources are transformed to make them directly comparable [11, 12]. When dealing with mental health questionnaires, one approach is to harmonise at the question/item-level. Although questionnaires can differ considerably on the number and nature of questions asked, there is often overlap in the semantic content of certain questions (see Table 1 for an example of similar items from two different measures). By identifying conceptually similar item-pairs, researchers can construct bespoke harmonised subscales that can be used for cross-study analyses.

**Table 1:** Example of similar items from two different scales

Moods and Feelings Questionnaire [13]		CES-D [14]	
Question	**Content**	**Question**	**Content**
**10**	I felt lonely	14	I felt lonely
**1**	I felt miserable or unhappy	18	I felt sad
**12**	I thought I could never be as good as other people	4	I felt I was just as good as other people^a^
**7**	I found it hard to think properly or concentrate	5	I had trouble keeping my mind on what I was doing
**6**	I cried a lot	17	I had crying spells

^a^Reverse coding of responses would be required for analysis

Recent attempts to harmonise mental health questionnaires have largely relied on expert opinion to match items from different questionnaires [15, 16]. For instance, McElroy et al. [12] explored trends in child mental health using subsets of items from the Rutter Behaviour Scales [17] and the Strengths and Difficulties Questionnaire [18]. Two researchers screened the instruments independently of one another, and identified item-pairs they considered conceptually similar. Although inter-rater agreement was high (88%), a third independent rater served as the decision maker when the initial raters disagreed. This process produced a final harmonised subset consisting of seven questions that were consistent across the two scales (Table S1), and psychometric tests supported the equivalence of these items across four different studies.

**Fig. 1:**
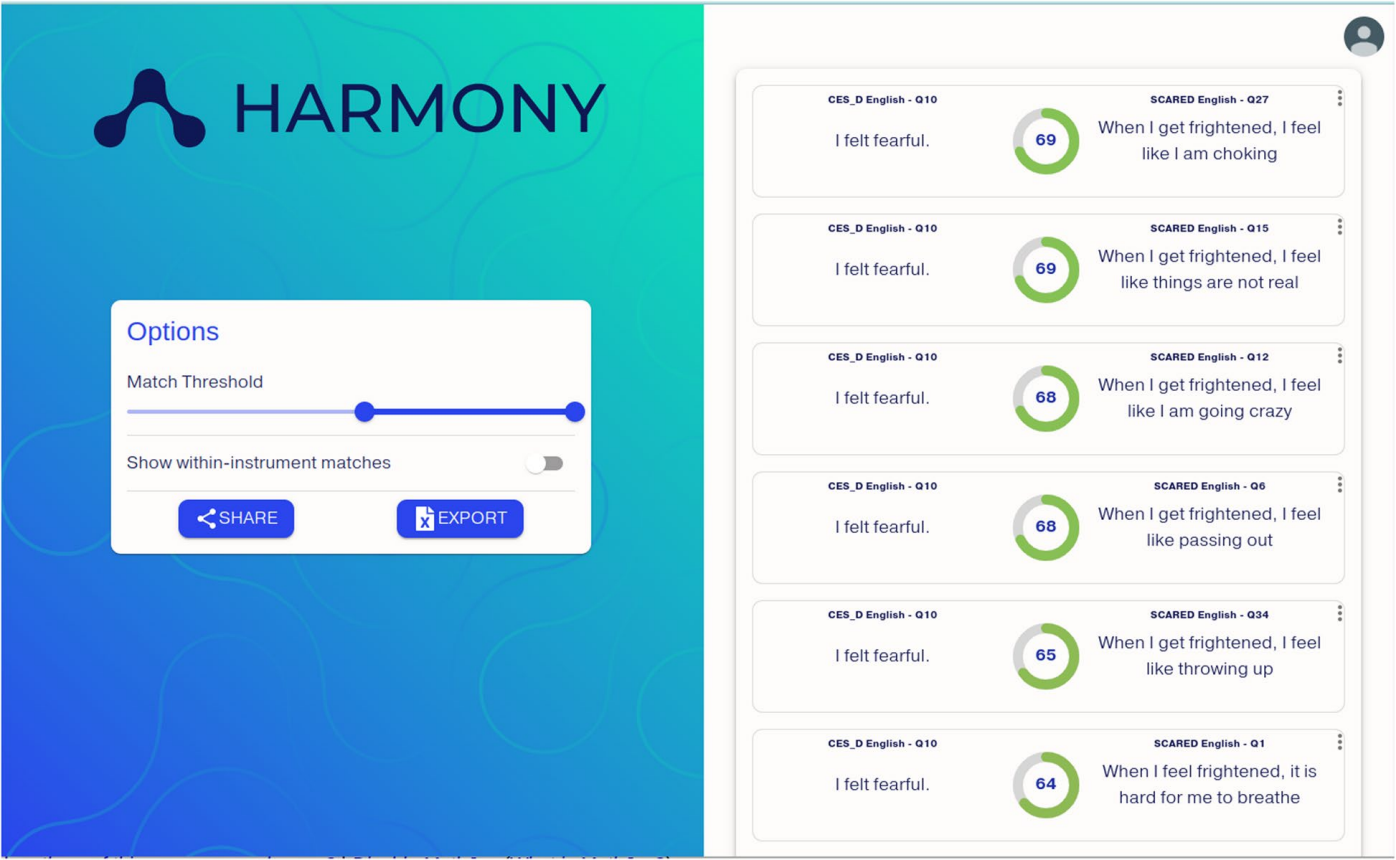
Screenshot of Harmony web interface. Cosine similarity indices presented in circles

Although the above results were promising, using expert opinion to match items has a number of inherent weaknesses. First, this approach relies on subjective ratings, and even when multiple raters are used, there will likely be some disagreement on which item-pairs should be included in the harmonised subscales. Second, manually harmonising questionnaires can become exponentially more challenging as the number of instruments increases. The rapid development and adaptation of natural language processing (NLP) technologies offers the chance to increase the speed, inter-rater reliability, and replicability with which questionnaires are retrospectively harmonised, and our research group has developed a free-to-use online tool, Harmony [19], for this purpose. Harmony (Fig. 1) is built in Python 3.10, and uses the Sentence-BERT model [20] to convert the text of each question within a scale into a unique numeric vector based on its semantic content. The similarity between two questions is then calculated as the distance between their respective vectors, expressed as the cosine similarity index (ranging from -1/ + 1, with values closer to 1 reflecting a greater semantic match).

While Harmony has the potential to be an important tool for the pooling of mental health data, it needs validation. If Harmony is producing valid matches (i.e. matching questions that describe conceptually similar experiences or behaviours), we would expect the strength of these matches to correspond with the degree to which subjects answer questions in the same way (i.e. the degree to which subject responses to items would correlate). Therefore, this exploratory study aims to quantify the association between the semantic item-matches produced by Harmony and item-pair correlations derived from real-world epidemiological data. We also explore Harmony’s ability to identify complex underlying structures (i.e. clusters of strongly related item-pairs) using a graph theory approach. Again, we compare the identified structure to that found in real-world item-wise correlational data.

## Methods

### Data

For our correlation analyses, we drew on data from Wave 6 of the COVID-19 Psychological Research Consortium (C19PRC) study [21]. This study began in March 2020 with the aim of monitoring the psychological, social and economic impact of the COVID-19 pandemic in the UK. The study initially comprised a nationally representative sample of 2,025 adults, with ‘top-up’ participants added at later waves. The sixth wave of data collection occurred between August and September 2021. At this sweep, 1,643 participants from earlier waves were re-interviewed, and an additional 415 new respondents were surveyed (*N* = 2,058) and the final sample matched the original sample in terms of the quota-based sampling. All participants had complete data. The mean age of participants was 45.92 years (SD = 15.79), 51.9% of the sample were female, 87.7% of the sample were of white British/Irish ethnicity, 57.6% had post-secondary education, and 64.2% were in either full-time or part-time employment. Wave 6 of the C19PRC study was granted ethical approval by the University of Sheffield [Reference number 033759]. The data and meta-data used in this study can be found at https://osf.io/v2zur/.

### Measures

We drew on data from five self-report questionnaires. Two of these questionnaires assessed depression, two covered anxiety, and one measured symptoms of PTSD.

The Patient Health Questionnaire-9 (PHQ-9) [22] consists of nine questions that align with the DSM-IV criteria for major depressive disorder. Participants were asked about the frequency with which they experienced these depressive symptoms over the preceding two weeks. Response options were on a 4-point Likert scale ranging from 0 (not at all) to 3 (nearly every day). The psychometric properties of the PHQ-9 have been extensively documented [23].

Participants also completed the Generalized Anxiety Disorder Scale (GAD-7) [24]. Respondents were asked to indicate on a 4-point Likert scale ranging from 0 (not at all) to 3 (nearly every day), how frequently they were troubled by seven symptoms of anxiety over the preceding two weeks. The reliability and validity of the GAD-7 has been supported widely evidenced [25].

Two newly developed scales were also administered; the International Depression Questionnaire (IDQ) and the International Anxiety Questionnaire (IAQ) [26]. These scales were designed to align with the ICD‐11 descriptions of Depressive Episode and Generalized Anxiety Disorder. The IDQ consists of nine questions, and the IAQ has eight. For both questionnaires, responses are indicated on a 5-point Likert scale ranging from 0 (Never) to 4 (Every day). Initial psychometric work suggests these scales are reliable and valid [26].

The International Trauma Questionnaire (ITQ) [27] was used to screen for ICD-11 post-traumatic stress disorder (PTSD). The ITQ consists of six questions that can be grouped into two-item symptom clusters of Re-experiencing, Avoidance, and Sense of Threat. Participants were asked to complete the ITQ as follows: “…in relation to your experience of the COVID-19 pandemic, please read each item carefully, then select one of the answers to indicate how much you have been bothered by that problem in the past month”. Responses were indicated on a 5-point Likert scale, ranging from 0 (Not at all) to 4 (Extremely). Three additional questions measure functional impairment caused by the symptoms. The psychometric properties of the ITQ scores have been supported in both general population [28] and clinical and high-risk [29] samples.

All 39 questions from the five scales, which were used as input for our NLP analyses, are presented in the supplementary files (Table S2). All questions were scored in the same direction (i.e. higher scores reflected greater frequency/severity of symptomatology), therefore no reverse coding was required.

### Pre-processing

First, using the data from the C19PRC study, we calculated a Spearman rank correlation for each pair of questions in the battery. Given there were 39 questions in total, this resulted in 741 correlation coefficients (39*38/2). Second, we imported the questionnaire content, in pdf format, into Harmony, which produced a semantic similarity score (cosine index) for each of the 741 item-pairs. We then merged the results from the above two steps, creating a simple data set where the rows corresponded to item-pairs, and columns corresponded to correlation and cosine values for each item-pair (available in Supplementary file 2).

### Analyses

We explored the association between the correlations from the empirical data and NLP-derived similarity scores by doing the following:

First, we randomly split the dataset into training (80% of item-pairs) and testing samples (20% of item-pairs). Using the training sample, we produced a scatterplot to visualise the relationship between the cosine and correlation scores, and then calculated the Pearson correlation between the two indices. Next, we estimated a linear regression model, with cosine scores as the predictor and correlation coefficients as the outcome variable. We then tested this model in the holdout sample, and calculated the mean absolute error (MAE), and Root Mean Squared Error (RMSE), the Median Absolute Error (MedAE) between what was predicted by our model and the observed correlation coefficients in the holdout sample. These errors were visualised as a violin plot. All of the above analyses were conducted in R version 4.3.1 and visualisations were produced using the ggplot2 package [30].

Next, to examine the ability of NLP to uncover complex structures using questionnaire meta-data, we estimated and visualised matrices of the item-pair correlations and cosine scores as separate graphical networks using the full dataset (*N* = 741). In the network of cosine scores, nodes (points in space) represented questions, and edges (connections between nodes) reflected the cosine similarity scores between a given pair of questions, with thicker and more saturated lines indicating higher cosine values. We estimated two versions of the correlation network – a bivariate/pairwise correlation network, and a regularised partial correlation network. In the bivariate network, nodes represented questionnaire variables and edges reflected the strength of the correlations between nodes. In the regularised partial correlation network, edges can be interpreted as partial correlation coefficients, with line thickness and saturation reflecting the strength of association between two symptoms after controlling for all other symptoms in the network. In this network, a LASSO penalty was applied to the edges, which shrinks edges and sets very small connections to zero. This is a commonly employed approach in the estimation of networks of mental health data, as it produces a sparse network structure that balances parsimony with explanatory power [31]. The LASSO utilizes a tuning parameter to control the degree of regularization that is applied. This is selected by minimizing the Extended Bayesian Information Criterion (EBIC). The degree to which the EBIC prefers simpler models is determined by the hyperparameter γ (gamma) – this was set to the recommended default of 0.5 in the present study [31]. For further detailed information on the estimation of regularised partial correlation networks, we refer readers elsewhere [31, 32]. The networks in the present study were estimated and visualised in R using the qgraph package [33].

After estimating the cosine and correlation networks, we used the walktrap community detection algorithm [34] to identify communities or clusters of nodes within the three networks. Walktrap is a bottom-up, hierarchical approach to uncovering structures within networks. The central idea of walktrap is to simulate random walks within a given network. Random walks start from a particular node and traverse the network by moving to a neighboring node at each step, following edges randomly. This process is repeated for multiple random walks initiated from each node in the network. Walktrap is based on the idea that nodes within the same community will have similar random walk patterns and thus be close to each other in the clustering. We ran the walktrap algorithm using the igraph package, taking the weighting of edges into account, with the default number of four steps per random walk. Research has shown that the walktrap method produces similar results to other methods for uncovering underlying structures in multivariate data (e.g. exploratory factor analysis, parallel analysis) [35]. However, the walktrap algorithm can produce a clustering outcome, even in scenarios involving entirely random networks. Consequently, we calculated the modularity index *Q* [36] to assess the clarity and coherence of the clustering solutions identified. In real-world data, *Q* typically ranges from 0.3 to 0.7, with values closer to 0.3 indicating loosely defined communities, while those around 0.7 indicate well-defined and robust community structures [36].

While the LASSO networks offer a more conservative and interpretable structure than networks consisting of bivariate correlations, to our knowledge, there is no equivalent approach for networks of cosine scores. Furthermore, there are no guidelines for determining when two questions are considered ‘similar enough’ based on their cosine similarity score. To address this, we conducted sensitivity analyses in which we manually set small edges (cosine vales) to zero, to produce increasingly sparse networks. We estimated five additional cosine networks, removing any connections with edge weights below certain thresholds. These thresholds ranged from 0.2 up to 0.6. For each of these networks we also tested for community structures and modularity.

## Results

**Fig. 2:**
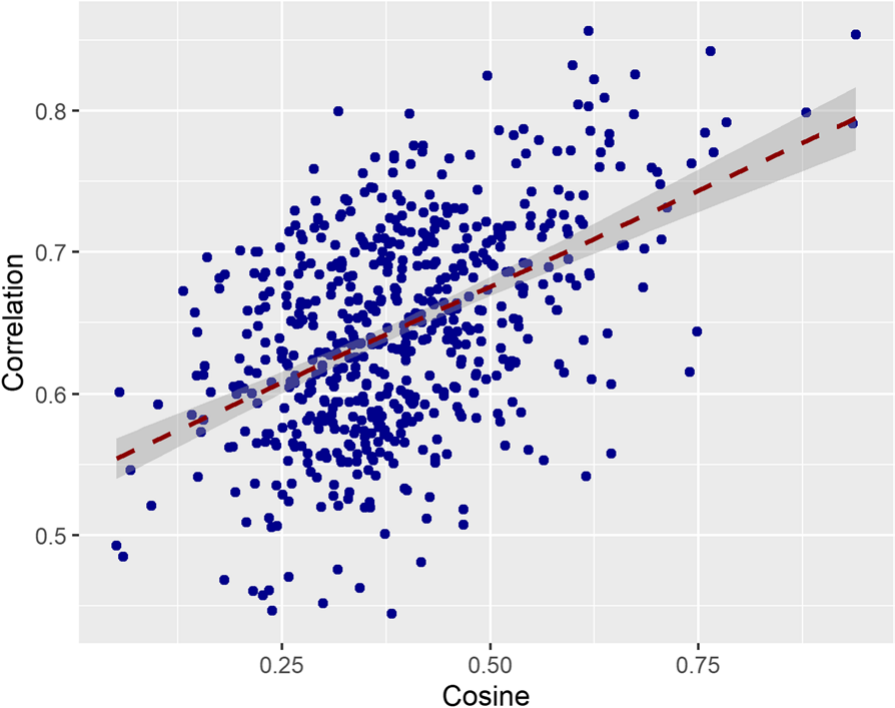
Scatterplot of cosine scores and Spearman correlation coefficients from item-pairs in the training sample (*N* = 592). Each dot represents the value of the cosine score (x-axis) and correlation coefficient (y-axis) of a specific item-pairing

The mean inter-item correlation coefficient in the training sample was *r* = 0.64 (SD = 0.07), and the mean cosine value was 0.39 (SD = 0.14). Distributions of these values are presented in Figure S1. The Spearman correlation coefficients appeared relatively normally distributed, whereas the cosine scores were slightly positively skewed. However, skewness and kurtosis values were in acceptable ranges (Figure S1). Figure 2 plots the cosine score and Spearman correlation coefficient of each of the question-pairs in the training sample. The correlation between the cosine and Spearman values was 0.48 (*p* < 0.001; 95% CI = 0.42—0.54), indicating a moderate correlation between the two values.

**Fig. 3:**
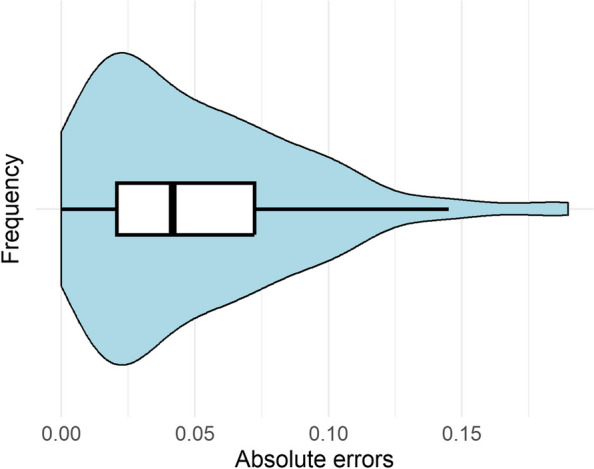
Violin plot of absolute error values between predicted and observed correlation scores in holdout sample (*N* = 149)

The Rainbow test for non-linearity was conducted, and confirmed that the linear regression model was appropriate for the data (*F* = 0.75; *p* = 0.99). In the linear regression model, cosine scores were a significant predictor of item-pair correlations (b = 0.27, a = 0.54, *R*^2^ = 0.23, F[1, 590] = 179.8, *p* < 0.01). Next, using the 20% holdout sample, we calculated the mean absolute errors (MAE) between what was predicted by our model and the observed correlation scores. The MAE was 0.05 (SD = 0.04), and the error values are visualised as a violin plot in Fig. 3. This indicates that when using the semantic similarity between items to predict the actual correlation between participant answers, the model will on average have an error of ± 0.05, which can be considered a minor error. We also calculated the Median Absolute Error (MedAE), and Root Mean Squared Error (RMSE), which are less sensitive to outliers. Both MedAE (0.04) and RMSE (0.064) also suggested a low level of error in our predictive model.

**Fig. 4:**
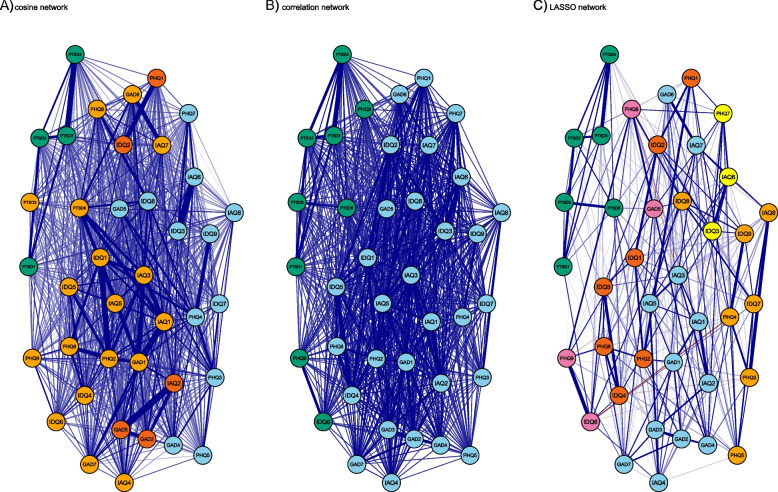
Cosine, correlation and LASSO networks. Cosine and LASSO networks based on full dataset (*N* = 741)

The cosine, bivariate and LASSO networks are presented in Fig. 4. As could be expected, the LASSO network was considerably more sparse than the other two. A full breakdown of the clustering of items (including exact question wording) is presented in Supplementary Tables 3, 4 and 5. In the bivariate correlation network, only two clusters were identified – a cluster dominated by PTSD and self-harm items, and a cluster consisting of the remaining items. In the cosine network, four clusters were detected. The first cluster consisted of the four avoidance and re-experiencing items from the ITQ. The second cluster captured several items related to worry and anhedonia. The third cluster included items related to sleep disturbances, fatigue, difficulty relaxing and concentrating. The final cluster of nodes captured a broad array of negative affectivity and psychological distress.

The LASSO network produced a 5-cluster solution. Cluster 1 (PTSD) consisted of all six items of the ITQ. Cluster 2 was formed of four items capturing restlessness and thoughts of self-harm. The third cluster was formed of three items capturing concentration problems. Cluster 4 included items that broadly related to feelings of negative affect (e.g. low mood, guilt, worthlessness, hopelessness). The fifth cluster tapped difficulties with sleep, appetite and fatigue. The final cluster had six items from the GAD-7 and six items from the IAQ and broadly captured general anxiety.

The modularity (*Q*) values were extremely low for the bivariate correlation (0.01) and cosine (0.04) networks. By contrast, the *Q* index in the LASSO network was 0.49, indicating a moderately well-defined and robust community structure. Networks, community structures and *Q* indices for the cosine networks with different threshold applied to the edges are presented in the Figure S2. The interpretability of clusters, along with modularity, increased considerably as smaller edges (i.e. cosine values) were set to zero in the networks. When cosine values of less than 0.5 were set to zero, the *Q* index was pushed above the 0.3 value, suggesting a non-random clustering of nodes. When setting the threshold at cosine values of 0.6 or greater, *Q* rose to 0.52, suggesting a well-defined community structure. However, when the threshold was set this high, many nodes did not have any connections to the broader network.

## Discussion

The present study aimed to test the degree with which NLP measures of semantic similarity were associated with correlations in real-world mental health questionnaire data. We found a moderate correlation between cosine similarity indices (produced by the Sentence-BERT model) and Spearman coefficients for the same item-pairs in a battery of 39 mental health questions. In our holdout sample, we found that the cosine score of an item-pair could predict the real-world correlation coefficient with a mean error of ± 0.05. These findings suggest that cosine scores can, with reasonable accuracy, predict bivariate correlations between pairs of mental health questionnaire items.

Our second aim was to explore whether NLP could uncover more complex structures underlying mental health questionnaire data. Low modularity in the cosine network coupled with the general inconsistency/vagueness of the clustering solution suggested that our NLP tool performed poorly in this regard. This was due to the high level of connectivity that was observed when all cosine scores were included in the network. This was in stark contrast to the LASSO network, which imposed a penalty on the smallest edges, and therefore had high modularity and produced interpretable and meaningful clusters. Indeed, when thresholds were applied to the edges included in the cosine networks, more clearly defined communities of nodes emerged, and modularity indices improved to the 0.3—0.7 range that deemed acceptable in real-world data [36]. While further research (e.g. simulation work) is required to determine how best to apply thresholds to cosine similarity indices in this context, our findings suggest that NLP methods offer promise in identifying clusters of related variables based solely on meta-data. As such, NLP may become a useful means of approximating correlations between mental health items and scales prior to expensive data collection.

Overall, our findings provide initial support for using NLP as a means of identifying candidate questionnaire items for retrospective harmonisation. However, it is important to note that simply identifying questions based on their semantic similarity does not guarantee the psychometric equivalence of measures. There are many sources of bias that can threaten the comparability of results across different data sources. For instance, methods of administration (e.g. online vs pen and paper) or differences in response options can influence how participants answer questionnaires [37]. Furthermore, different groups or populations may interpret questions differently or respond in systematically different ways [38]. Therefore, we recommend that researchers explicitly test the equivalence of conceptually similar items in their data before they are used for cross-study research. There are various methods commonly used for such purposes, such as item response theory (IRT) and multiple group confirmatory factor analysis (CFA). Broadly, these approaches estimate latent variable measurement models of a given construct (e.g. depression) in two or more groups (e.g. samples from two different studies). Increasingly stringent equality constraints are then placed on the measurement parameters in the two groups, which are used to test the plausibility that the items are equivalent and therefore meaningful comparisons can be made across groups [38]. However, it is also worth noting that NLP models are developing rapidly, therefore the accuracy with which they could be used mirror real-world correlations could be expected to increase.

Our findings suggest there is immense potential for NLP to influence various areas of questionnaire based research. As demonstrated here, NLP could be used to identify candidate items for retrospective harmonisation. NLP tools such as Harmony could also be integrated with data discoverability tools to help researchers find and pool data from different sources. In addition, it would be relatively straightforward to adapt NLP models to facilitate scale development and validation; e.g. by identifying and comparing the semantic overlap of different pools of items.

### Strengths and limitations

The present study had a number of strengths. Our empirical data were drawn from a representative UK sample of adults. Our questionnaire battery contained overlapping measures (i.e. two measures of depression, two measures of anxiety), and these were completed by the same participants – meaning our data were well-suited for testing semantic similarity and inter-item correlations. In terms of limitations, the present findings, including our predictive model, generalise only to the present battery of items within a community sample. The use of Harmony in other areas of research would require further validation using a broader range of questionnaires and topics (e.g. wellness, personality). Similarly, further validation in clinical samples may be required if Harmony is to be used to pool data from clinical studies. Second, our study relied on commonly used measures that were developed in Western contexts – therefore it is unclear whether similar results would be produced across different languages and culturally sensitive questionnaires. Third, our findings are based solely on the Sentence-BERT model [20], and it is possible that alternative NLP models could produce different results. Fourth, although Harmony is sensitive to antonyms (i.e. sentences that convey opposite meanings are coded with negative cosines), further validation work is required to explicitly compare the tools ability to match antonyms and synonyms. However, recent research suggests that the BERT model is accurate at identifying antonyms [39].

## Conclusion

To the best of our knowledge, this is the first study to explore whether NLP methods can be used to match pairs of items from mental health questionnaires based on their semantic content, and whether these matches align with real-world inter-item correlations. Our findings indicate that NLP matches, expressed as cosine indices of similarity, can predict bivariate correlations with a reasonable degree of accuracy. Our NLP model was also able to identify more complex underlying structures within our data, however this required manual constraints to be placed on the edges that were included int the network, and therefore further research is required to establish best practices in this regard. Overall, these findings suggest that NLP can be a useful tool for researchers who wish to identify similar items for cross-study pooling of data. However, it remains important to explore the psychometric equivalence of candidate items.

### Supplementary Information

Supplementary Material 1.

Supplementary Material 2.

Supplementary Material 3.

## Data Availability

The data and meta-data from the C19PRC study can be found at https://osf.io/v2zur/. The correlation and cosine values used in the present analyses are available in Supplementary file 2.

## Abbreviations

C19PRC: COVID-19 Psychological Research Consortium
CFA: Confirmatory factor analysis
EBIC: Extended Bayesian Information Criteria
GAD-7: Generalised Anxiety Disorder Assessment
IAQ: International Anxiety Questionnaire
IDQ: International Depression Questionnaire
IRT: Item response theory
ITQ: International Trauma Questionnaire
LASSO: Least absolute shrinkage and selection operator
MAE: Mean absolute error
NLP: Natural language processing
PHQ-9: Patient Health Questionnaire-9
Sentence-BERT: Sentence Bidirectional Encoder Representations from Transformers
