# Supplementary material for: Using natural language processing to facilitate the harmonisation of mental health questionnaires: a validation study using real-world data
Source: BMC Psychiatry. 2024 Jul 24;24:530. doi: 10.1186/s12888-024-05954-2 (PMC11267737; doi:10.1186/s12888-024-05954-2)
Supplement: Supplementary file 1 — Supplementary Material 1. [file 12888_2024_5954_MOESM1_ESM.docx]

# **Using natural language processing to facilitate the harmonisation of mental health questionnaires: a validation study using real-world data**

Supplementary Materials

| Table S1. Harmonised emotional and behavioural problems sub-scales (adapted from McElroy et al., 2023) | | |
| --- | --- | --- |
|  | **Rutter Parent Questionnaire** | **SDQ** |
| *Core symptom* | ***Emotional problems*** |  |
| Low mood | 9. Often appears miserable, unhappy, tearful or distressed. | 13. Is often unhappy, down hearted or tearful |
| Worry | 6. Often worried, worries about many things | 8. Has many worries, often seems worried |
| Fear | 16. Tends to be fearful or afraid of new things or new situations. | 16. Is nervous or clingy in new situations, easily loses confidence |
|  | ***Behavioural problems*** |  |
| Physical aggression | 4. Frequently fights other children. | 12. Often fights with other children or bullies them |
| Disobedience | 14. Is often disobedient | 7. Is generally obedient, usually does what adults request* |
| Irritability | 8. Irritable. Is quick to fly off the handle. | 5. Often has temper tantrums or hot tempers |
| Lying | 17. Often tells lies | 18. Often lies or cheats |

*reverse coded

| **Table S2. Questionnaire items used in correlational and NLP analyses** | | |
| --- | --- | --- |
| **Questionnaire** | **Number** | **Content** |
| IDQ | 1 | Felt down or depressed for most of the day? |
| IDQ | 2 | Experienced less interest or pleasure from normal activities for most of the day? |
| IDQ | 3 | Have had difficulty concentrating? |
| IDQ | 4 | Had feelings of worthlessness or guilt? |
| IDQ | 5 | Felt hopeless? |
| IDQ | 6 | Had recurrent thoughts of death or suicide? |
| IDQ | 7 | Have had changes in appetite or sleep? |
| IDQ | 8 | Moved slower or felt more restless? |
| IDQ | 9 | Experienced reduced energy or fatigue? |
| IAQ | 1 | Felt nervous or anxious? |
| IAQ | 2 | Worried a lot about different things? |
| IAQ | 3 | Felt physically tense or agitated? |
| IAQ | 4 | Felt your heart racing, difficulty breathing, stomach discomfort, or dry mouth? |
| IAQ | 5 | Felt “on edge”? |
| IAQ | 6 | Had difficulty concentrating? |
| IAQ | 7 | Been easily annoyed by different things? |
| IAQ | 8 | Experienced sleep disturbances? |
| PHQ | 1 | Little interest or pleasure in doing things? |
| PHQ | 2 | Feeling down, depressed, or hopeless? |
| PHQ | 3 | Trouble falling or staying asleep, or sleeping too much? |
| PHQ | 4 | Feeling tired or having little energy? |
| PHQ | 5 | Poor appetite or overeating? |
| PHQ | 6 | Feeling bad about yourself - or that you are a failure or have let yourself or your family down? |
| PHQ | 7 | Trouble concentrating on things, such as reading the newspaper or watching television? |
| PHQ | 8 | Moving or speaking so slowly that other people could have noticed? Or the opposite - being so fidgety or restless that you have been moving around a lot more than usual? |
| PHQ | 9 | Thoughts that you would be better off dead, or of hurting yourself in some way? |
| GAD | 1 | Feeling nervous, anxious or on edge? |
| GAD | 2 | Not being able to stop or control worrying? |
| GAD | 3 | Worrying too much about different things? |
| GAD | 4 | Trouble relaxing? |
| GAD | 5 | Being so restless that it is hard to sit still? |
| GAD | 6 | Becoming easily annoyed or irritable? |
| GAD | 7 | Feeling afraid as if something awful might happen? |
| ITQ | 1 | Having upsetting dreams that replay part of the experience or are clearly related to the experience? |
| ITQ | 2 | Having powerful images or memories that sometimes come into your mind in which you feel the experience is happening again in the here and now? |
| ITQ | 3 | Avoiding internal reminders of the experience (for example, thoughts, feelings, or physical sensations)? |
| ITQ | 4 | Avoiding external reminders of the experience (for example, people, places, conversations, objects, activities, or situations)? |
| ITQ | 5 | Being “super-alert”, watchful, or on guard? |
| ITQ | 6 | Feeling jumpy or easily startled? |


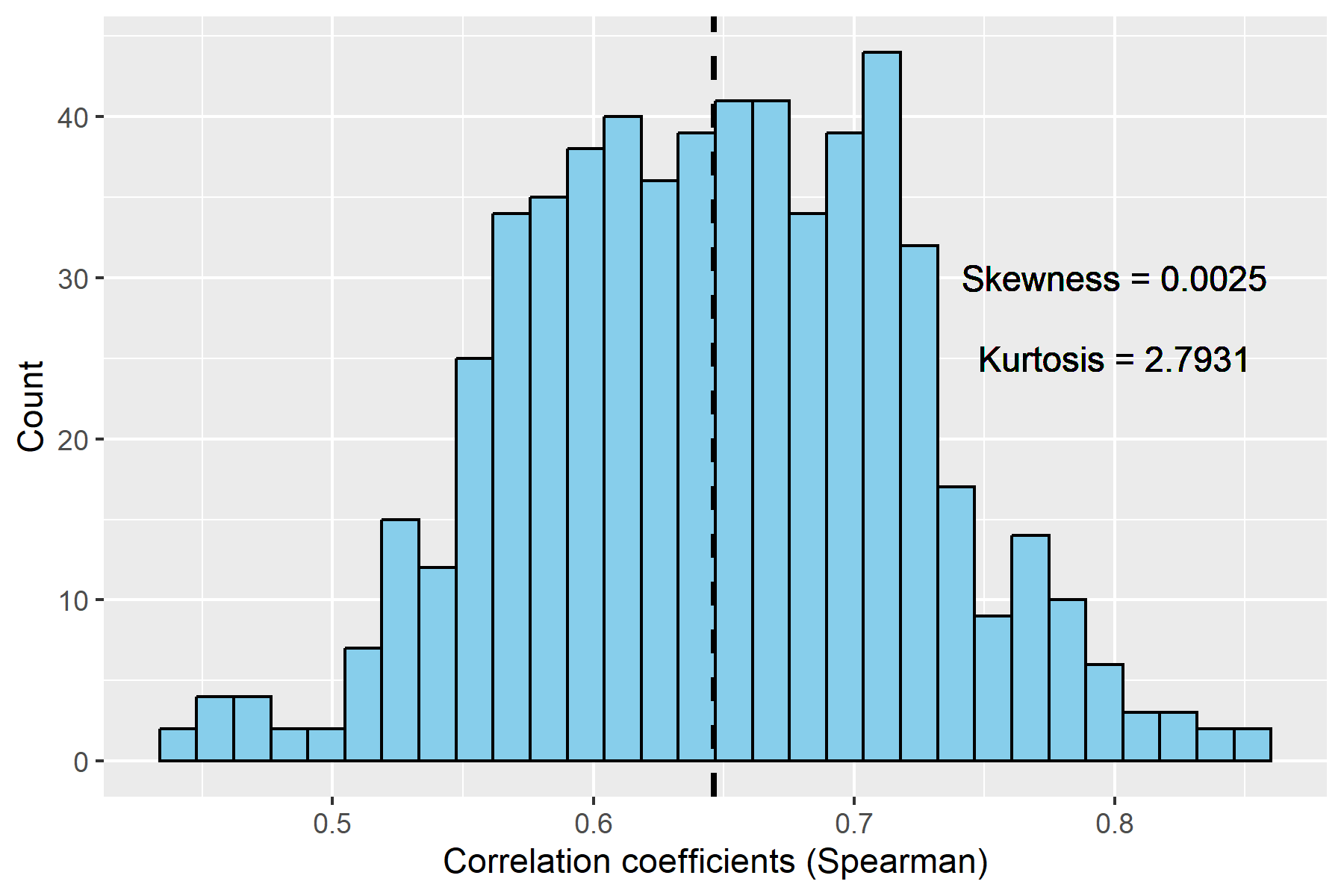

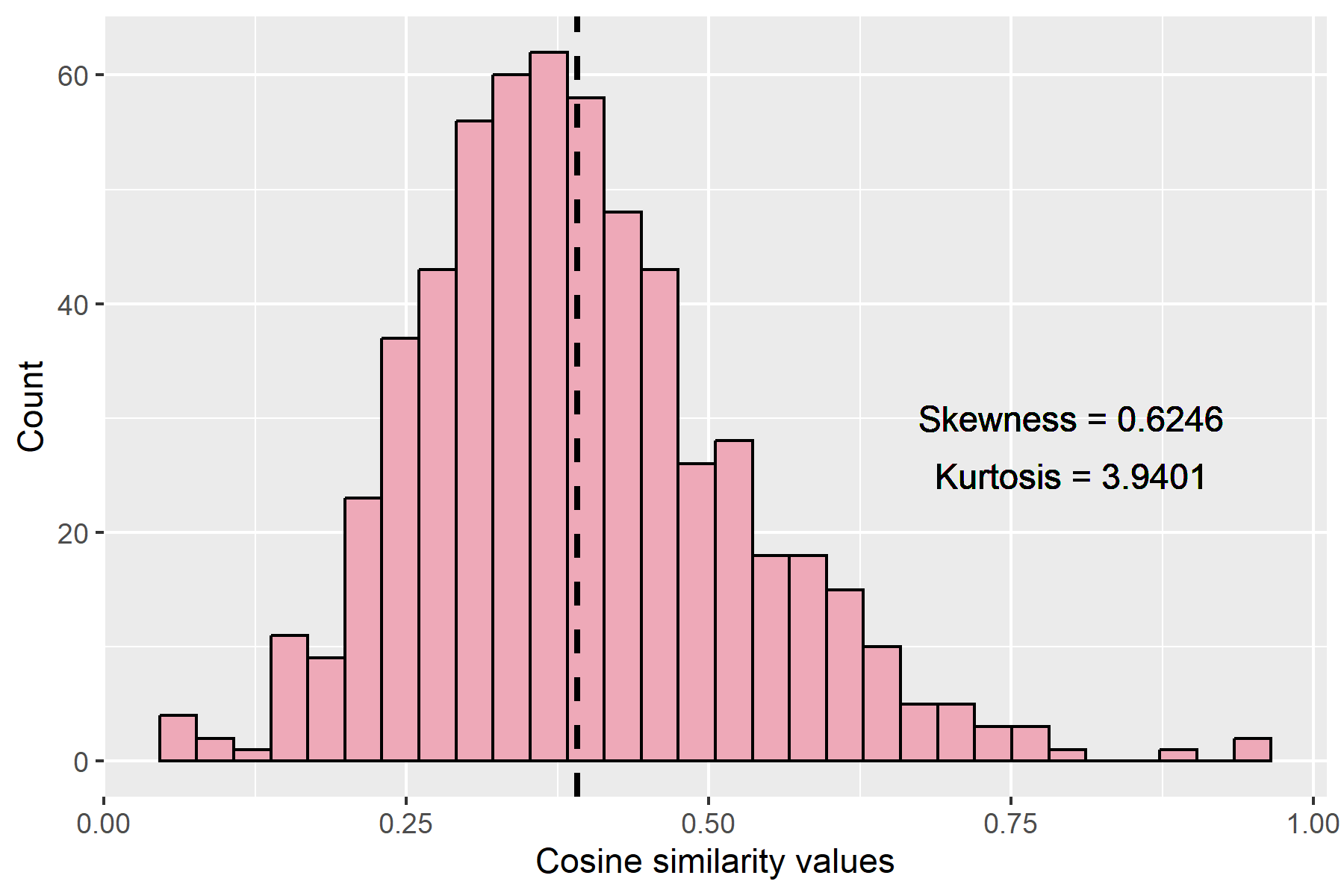


Figure S1.Distributions of Spearman correlation coefficients (left) and cosine similarity scores (right) for all 749 item-pairings


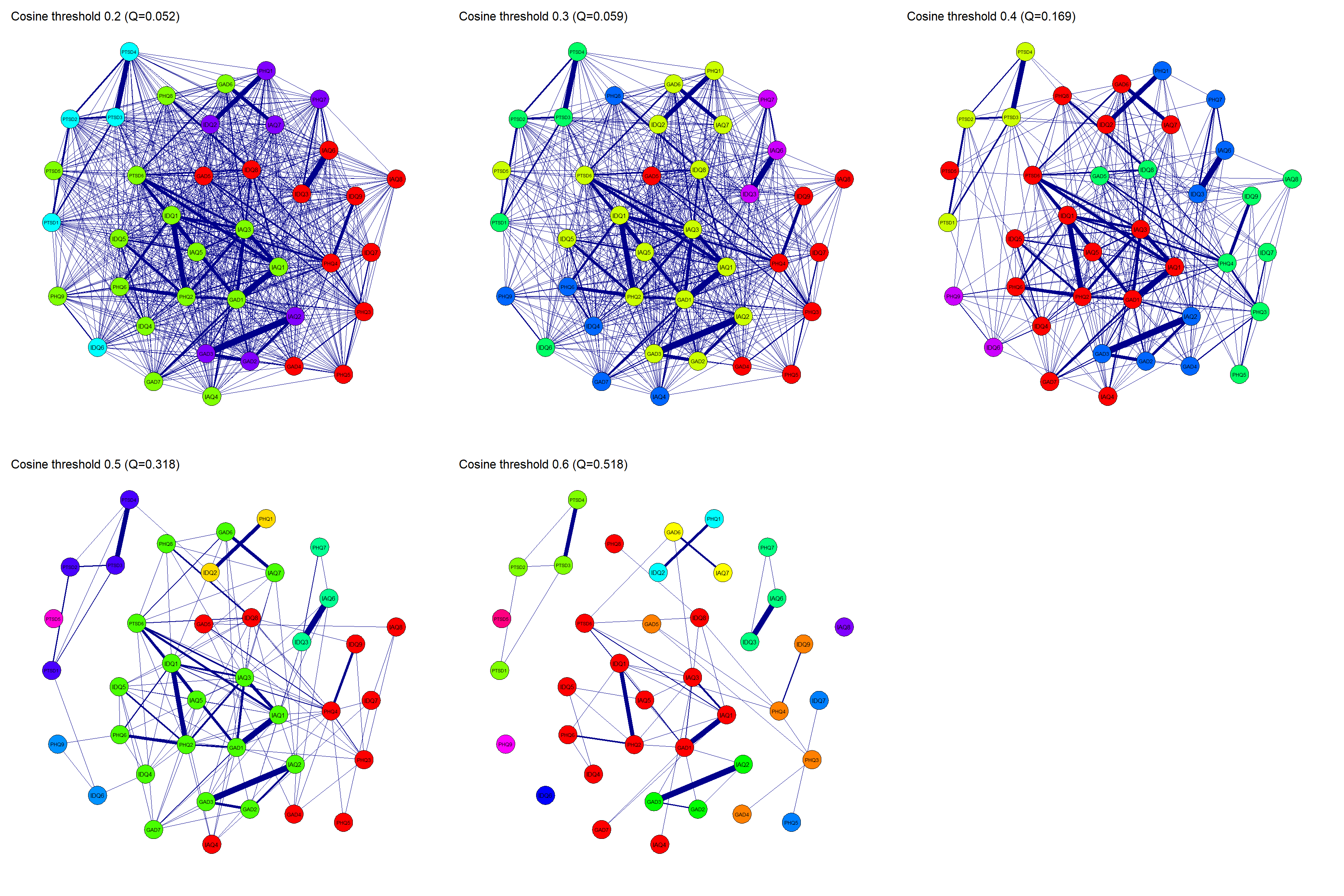


Figure S2. Cosine networks with increasingly stringent cut-offs applied to included edges
